# Legislation

**Published:** 1885-03

**Authors:** 


### Legislation.

The dentists of Dakota Territory are moving, and with considerable prospect of success, for the procuring of a law to regulate the practice of dentistry. This is certainly very gratifying to every one interested in the success of the profession, as a whole. Minnesota is also making an effort for the accomplishment of the same purpose.

It is very encouraging that the profession in these newer parts of our country realize the importance of such legislation as will secure to the public immunity from the practices of charlatans and quacks, and give encouragement and stimulus to those who will pursue the practice of the profession legitimately.

Quackery not only does incalculable mischief to the public, but degrades any profession in which it exists. In this respect dentistry has been unfortunate. It is only within the last third of a century that it has assumed such proportions and status, as entitle it to be regarded as a profession, or even a branch of a profession. It has not the prestige of age that is accorded to law, medicine, and surgery. It has had hanging to it a millstone of quackery. This, the profession is now asking the people of the several States to help remove, and this aid is promptly accorded whenever there is a reasonable amount of intelligence on the subject.

States that are now seeking such enactments are asking for more stringent and effective laws than were enacted twelve or fifteen years ago, when such proceding was regarded, even by the most sanguine, as but experimental, and by many was deemed as wholly utopian. The benefit of such laws having been clearly shown, and even by those defective in some respects, there is now a manifest inclination to secure such laws by the profession in almost every State in the Union.

Not only is it important that every State should have a law to regulate the practice of dentistry, but those States that have laws that are defective or inefficient, should seek to have them amended at the earliest possible period.

